# Leveraging network pharmacology in the treatment of asthma

**DOI:** 10.5599/admet.3281

**Published:** 2026-05-07

**Authors:** Sarthi Ahuja, Richard C. Kashindye, Divya Yadav, Priyanka Chaudhary, Rakesh Yadav

**Affiliations:** 1 School of Pharmacy, National Forensic Science University, Gandhinagar, Gujarat, 382 007, India; 2 SGT College of Pharmacy, SGT University, Gurugram, 122 505, India

**Keywords:** Molecular dynamics simulation, molecular docking, herbal medicine, systems pharmacology, multi-target therapy

## Abstract

**Background and purpose:**

Asthma, a chronic airway inflammatory disorder driven by multifaceted genetic, cellular and molecular interactions, remains inadequately managed by single-target therapies due to incomplete disease control and adverse effects; this review aimed to explore network pharmacology's role in elucidating multi-target mechanisms of phytotherapeutic agents for asthma, with a focus on integrating ADMET/DMPK profiling to predict clinical translatability and safety.

**Experimental approach:**

We employed network pharmacology methodologies including target prediction (*e.g. via* PharmMapper, PubChem), protein-protein interaction network construction (STRING, Cytoscape), pathway enrichment analysis (KEGG, Reactome), molecular docking (AutoDock) and ADMET/DMPK modelling (SwissADME, pkCSM) to dissect multi-component herbal formulations, complemented by literature-mined experimental validations.

**Key results:**

Analyses identified key asthma-related targets (*e.g.* IL-17, TNF) and pathways (JAK-STAT, PI3K-AKT), revealing quercetin and kaempferol's multi-target efficacy in reducing airway inflammation and immune dysregulation; favourable ADMET profiles (high oral bioavailability, low toxicity) and DMPK parameters (metabolic stability *via* CYP inhibition) supported their therapeutic potential in herbal combinations.

**Conclusion:**

Network pharmacology, enhanced by ADMET/DMPK integration, advances a holistic understanding of herbal asthma treatments, promoting safe, multi-target drug development. Limitations include data gaps in multi-omics validation and herbal standardization, with future directions leveraging AI-driven predictions for personalized pharmacotherapy.

## Introduction

Asthma is a heterogeneous, long-term inflammatory airway disease, a pathological condition characterized by a spectrum of clinical manifestations, including wheezing, dyspnea, chest constriction and a persistent cough, coupled with a restriction in expiratory airflow. It remains a major global health challenge affecting all age groups, with disease burden varying across regions, genders and socioeconomic strata, and disproportionately impacting low-income populations [1]. In 2021, asthma was responsible for an estimated 260.48 million prevalent cases, 37.87 million newly diagnosed cases, 436.19 thousand fatalities and 21.42 million disability-adjusted life years (DALYs) worldwide. South Asia bore the highest burden, contributing 45.12 million prevalent cases, 7.69 million incident cases, 348.21 thousand deaths and 9.87 million DALYs [2]. India contributes dispropare moreonately to the global asthma burden, with 34.3 million cases (13.09 % of the global total) and mortality and DALY rates more than two-fold higher than global averages [3]. A pronounced urban-rural disparity exists, with urban prevalence reported at 45 % compared to rural regions and asthma showing a higher prevalence among individuals from higher socioeconomic backgrounds in India [4]. Current asthma management relies predominantly on synthetic pharmacological agents, including antihistamines, decongestants, expectorants, mucolytics, bronchodilators, cough suppressants and biologic therapies [5-6]. Inhaled corticosteroids (ICs), either alone or in combination with long-acting β₂-adrenergic agonists (LABA), remain the cornerstone of therapy [7].

Although these treatments effectively control airway inflammation and bronchoconstriction, their long-term clinical utility is limited by several adverse outcomes, including glucocorticoid-associated complications, increased susceptibility to infections, metabolic disorders, neoplasms, worsening asthma and nasopharyngitis, as well as a substantial economic burden [6,8]. These safety and tolerability concerns significantly impair patient compliance and long-term therapeutic outcomes. The limitations associated with conventional therapies have intensified the need for safer, cost-effective and better-tolerated alternatives, thereby driving growing interest in herbal and natural product-based interventions for asthma management (8).

Herbal and natural product-based therapies are increasingly being explored as supportive options in asthma management, particularly in combination with conventional treatment [9-10]. Across many traditional medical systems, plant-derived remedies have long been used for respiratory conditions, and their therapeutic effects are rarely attributed to a single compound [11]. Instead, most herbal preparations contain multiple bioactive constituents that may act on different biological processes simultaneously. Findings from experimental models and clinical observations suggest that such formulations can alleviate airway inflammation, influence immune responses and reduce oxidative stress, often with acceptable tolerability [12-13]. At the same time, the presence of numerous interacting components complicates mechanistic interpretation, as these effects cannot be readily explained using classical single-target pharmacological models. As a result, the molecular basis of many herbal interventions in asthma remains only partially understood, highlighting the need for analytical strategies capable of capturing multi-component and multi-pathway interactions, such as network pharmacology [14-17].

Network pharmacology provides a comprehensive systems-level framework that effectively captures the intrinsic complexity of asthma by elucidating interactions among bioactive compounds, disease-associated genes and interconnected signalling pathways, thereby enabling a more comprehensive understanding of disease modulation. In contrast to the traditional “one drug-one target” paradigm that underpins most conventional therapies, this approach emphasizes multi-compound and multi-target interactions, making it particularly suited to decoding the therapeutic potential and synergistic mechanisms of herbal medicines. Consequently, network pharmacology provides a rational and integrative strategy for identifying novel mechanisms of action and multi-pathway interventions relevant to asthma management [7].

Many studies have employed network pharmacology to explore herbal medicines in asthma; however, these findings are scattered across the literature and are rarely examined together. As a result, a unified understanding of how multi-component herbal interventions act through multiple molecular targets in asthma is still incomplete.

This review, therefore, aims to critically evaluate the use of network pharmacology to elucidate the molecular mechanisms underlying asthma management, with a particular focus on herbal and natural product--based therapies. The present review surveys network pharmacology investigations of asthma that utilize phytoconstituent-target identification, protein-protein interaction (PPI) networks, enrichment analyses and docking-based validation. Where available, evidence from experimental validation is also considered. Viewing these studies as a whole reveals a pattern in molecular targets and signalling pathways that are relevant to asthma, offering guidance for the development of improved multi-target treatment strategies.

## Principles of network pharmacology

Network pharmacology is a systemoriented approach that integrates polypharmacology and network biology to study drug-disease interactions at the level of biological networks rather than individual molecular targets. It recognizes that complex diseases such as asthma arise from dysregulation of interconnected signalling pathways and regulatory networks, rather than from isolated gene or protein defects. By mapping relationships among bioactive compounds, their molecular targets and downstream biological pathways, this approach provides a framework for understanding how multicomponent agents, such as herbal extracts, exert synergistic effects across multiple nodes within the disease network. In contrast to the classical “onedrug-onetarget” paradigm, network pharmacology embraces multitarget regulation, in which a single compound or a mixture (*e.g.* herbal formulation) may simultaneously modulate several proteins, thereby shifting the overall state of the pathological network toward a healthier configuration. This is particularly suitable for herbal medicines, which contain numerous phytochemicals that collectively influence complex disease phenotypes. By combining systems medicine with information science, network pharmacology enables systematic identification of key pharmacological targets, enrichment of relevant biological processes and signalling pathways, and prioritization of candidate compounds for further experimental validation [18,19].

Operationally, network pharmacology follows a structured workflow in which bioactive compounds are first identified and pharmacokinetically screened (for example, using oral bioavailability and druglikeness criteria) to retain only those with a higher likelihood of systemic exposure [7,20]. Targets of the screened compounds are predicted using *in silico* tools and literature evidence and then intersected with disease--related genes. Compound-target and protein-protein interaction (PPI) networks are constructed and analysed using networktopology metrics such as degree, betweenness centrality and closeness centrality to identify hub genes and critical regulatory nodes [21]. Functional enrichment analysis, such as Gene Ontology and Kyoto Encyclopedia of Genes and Genomes (KEGG) [22,23] pathway mapping, translates these network features into biological mechanisms, while molecular docking and, where feasible, experimental validation confirm the plausibility of predicted compound-target interactions at the structural and functional levels [7,21].

In the context of asthma, where multiple inflammatory, remodelling and immuneregulatory pathways interact dynamically, network pharmacology offers a rational strategy for deconvoluting the complex pharmacology of herbal extracts, identifying key modulated targets and pathways and guiding the design of more targeted and effective therapeutic interventions [20,24]. By integrating computational predictions with pharmacokinetic and pharmacodynamic considerations, this approach supports the discovery of novel antiasthmatic mechanisms and helps prioritize highvalue compounds for focused preclinical and Drug Metabolism and Pharmacokinetics (DMPK) studies [19].

## Network pharmacology as a systems approach to asthma.

**Figure 1.**
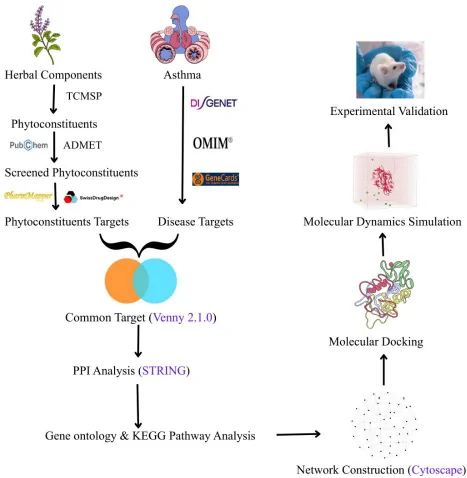
General workflow of network pharmacology

Network pharmacology integrates polypharmacology and network biology to decode drug-disease interactions at a systems level, moving beyond single-target paradigms. It maps bioactive compounds to molecular targets and pathways, revealing how multi-component herbal formulations rich in bioactive compounds modulate complex diseases like asthma through synergistic effects. This approach suits asthma research, where pathways like mitogen-activated protein kinase (MAPK) and Janus kinases - signal transducers/ /activators of transcription (JAK-STAT) drive inflammation, by identifying multi-target interventions informed by ADME feasibility. In our study, we applied a structured workflow (Figure 1) combining systems biology with pharmacokinetics to screen herbal compounds for asthma [14,25].

### Common network pharmacology workflow in asthma research

#### Compound identification and ADME screening

To align with the network pharmacology objective of identifying biologically relevant multicomponent agents, candidate herbal compounds were first retrieved from the Traditional Chinese Medicine Systems Pharmacology (TCMSP) database, PubChem and relevant literature on antiasthmatic herbal formulations [26,27]. These compounds were then subjected to oral bioavailability (OB) ≥ 30 % and druglikeness (DL) ≥ 0.18 filtering using the inbuilt pharmacokinetic predictors in TCMSP, which prioritized constituents with a higher likelihood of systemic exposure. This ADMEdriven triage ensured that only pharmacokinetically feasible compounds were carried forward into downstream target and network analyses [7].

#### Target identification

For the ADMEfiltered compounds, potential molecular targets were predicted using SwissTargetPrediction, PharmMapper and the Search Tool for Interacting Chemicals (STITCH) database [28,29,30], supplemented by manual extraction of reported targets from published literature. Concurrently, asthmaassociated genes were compiled from GeneCards, DisGeNET, Online Mendelian Inheritance in Man (OMIM), DrugBank and the Therapeutic Target Database (TTD) [31-35]. The intersection of compound-related targets and asthma-related genes was identified using Venn diagram analysis, yielding a core set of candidates, “compound-disease” targets, that were used as the basis for subsequent network construction [36].

#### Building and evaluating networks

Using the intersected target list, a compound-target-path Joi network was constructed in Cytoscape and PPI network was obtained from the STRING database [37]. The resulting networks were analysed using network topology metrics, such as degree, betweenness centrality and closeness centrality, to identify hub nodes. These hubs were considered putative key regulators within the asthma-herbal interaction network and were carried forward into functional enrichment and docking analyses [38].

#### Functional enrichment analyses

The hub targets were subjected to Gene Ontology (GO) and KEGG pathway enrichment analysis using an online bioinformatics tool (*e.g.* Database for Annotation, Visualization, and Integrated Discovery (DAVID)) [39]. GO terms were categorized into biological processes, molecular functions and cellular components, whereas KEGG analysis highlighted specific signalling pathways potentially modulated by the herbal compounds. This step explicitly linked the topological features of the network (hub genes) to functional mechanisms underlying asthma pathophysiology [40].

#### Docking-based validation

**Figure 2.**
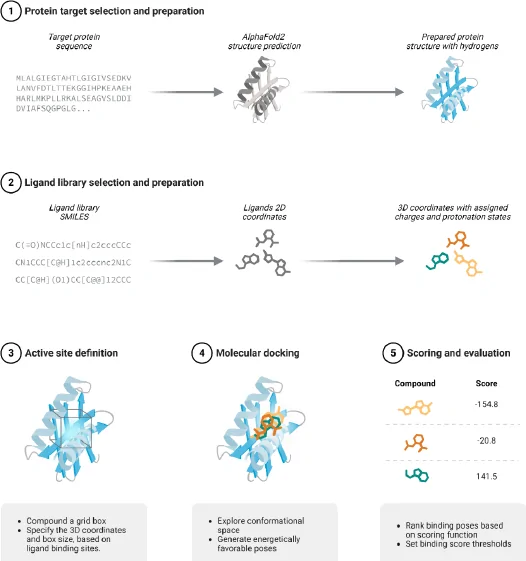
Workflow of molecular docking

To validate the predicted compound-target interactions at a molecular level, we performed molecular docking simulations between the top bioactive compounds and prioritized hub proteins using AutoDock Vina, Python Prescription (PyRx) or Molecular Operating Environment (MOE) as represented in Figure 2 [41].

Docking scores and interaction patterns (*e.g.* hydrogen bonds, hydrophobic contacts) were analysed to assess binding affinity and stability. In selected cases, these *in silico* findings were further supported by experimental validation using cell-based assays or *in vivo* models, thereby bridging network pharmacology predictions to pharmacological relevance [42].

By integrating computational predictions with biological data, network pharmacology effectively guides experimental validation by prioritizing key compounds, predicting relevant targets and suggesting appropriate biological readouts for testing. Furthermore, it facilitates novel drug discovery by uncovering new therapeutic molecules and signalling pathways that may extend beyond asthma.

## Molecular targets and signalling pathways in asthma explored by network pharmacology

Network pharmacology studies consistently identify key molecular targets involved in asthma pathogenesis. Prominent targets include Interleukins (ILs) like IL-17, IL-4, IL-13 and IL-6, pro-inflammatory factors like tumour necrosis factor (TNF), Signal Transducers and Activators of Transcription (STAT3), AKT1, Vascular Endothelial Growth Factor A (VEGFA) and Epidermal Growth Factor Receptor (EGFR). These molecules participate in the modulation of inflammatory and immune responses, which are critical for asthma development [7,24,38,40].

Signalling pathways commonly enriched in asthma network pharmacology analyses include the IL-17 signalling pathway, which is involved in neutrophilic inflammation and airway remodelling [20]. The TNF signalling pathway is central to inflammatory cytokine production and cellular recruitment [20,38,43,44]. HSP90AB1 protein is associated with the immunological response, as it can stimulate macrophages to produce inflammatory mediators (IL-6 and TNF-alpha). The processes of airway remodelling, excessive mucus secretion and the immune responses elicited by airway inflammation are all influenced by EGFR and VEGFA [7].

### Pathways in asthma

#### Akt kinase signalling axis

Phosphoinositide 3-kinase (PI3K) signalling cascade is profoundly linked to type 2 immune response and Th2-driven pulmonary response, which subsequently facilitates collagen accumulation, ultimately resulting in airway remodelling. The allergic asthma phenotype is elicited by sensitization to environmental allergens and is primarily mediated by Th2 cells, type 2 innate lymphoid cells (ILC2s), eosinophils, mast cells and immunoglobulin E (IgE). Conversely, intrinsic asthma arises independently of allergic stimuli and is associated with dysregulated inborn immunity driven by a multitude of factors, including pathogen invasion, adiposity, nicotine consumption and ecological toxins. This specific variant of asthma is distinguished by the participation of Th17 cells, ILC3s and neutrophils. The fundamental pathophysiological mechanisms underlying asthma involve the stimulation of immune cells, which subsequently elicit responses from non-immune cells, such as airway smooth muscle (ASM) and airway epithelial cells, culminating in AHR, inflammation and remodelling. The phosphoinositide 3-kinase (PI3K)/Akt signalling cascade plays a critical role in regulating a myriad of biological processes, including cellular proliferation, differentiation and migration. The activation of PI3K may lead to a decline in histone deacetylase 2 (HDAC2), which facilitates the recruitment of inflammatory cells, particularly neutrophils and promotes the release of pro-inflammatory factors and ROS overload. This signalling cascade is essential for orchestrating the responses of airway immune cells and structural cells implicated in the pathophysiological processes associated with asthma [45].

#### Janus kinases - signal transducers/activators of transcription

Th2 cells are responsible for the production of interleukins IL-4, IL-5, IL-6, IL-10 and IL-13, whereas Th1 cells release IL-2, IFN-γ and TNF-β. Th2 cytokines, primarily IL-4, IL-5 and IL-13, orchestrate the core elements of asthmatic inflammation, including IgE class switching, mucin production and eosinophil recruitment and activation. Stat6 is stimulated by interleukin 4 (IL-4) through the stimulation of Janus kinase 1 (Jak1) and Janus kinase 3 (Jak3). Conversely, engagement of interleukin 12 (IL-12) with its receptor activates Janus kinase 2 (Jak2) and tyrosine kinase 2 (Tyk2), thereby leading to the phosphorylation of signal transducer and activator of transcription 4 (Stat4). The function of Stat4 is primarily associated with the differentiation of T helper 1 (Th1) cells, whereas Stat6 is integral to the development of T helper 2 (Th2) cells. STAT6 also promotes B cells to switch antibody production to IgE. It also regulates chemokines that recruit inflammatory cells to the airways and promotes mucus production by airway epithelial cells. The pathway also controls the polarization and expansion of Th2 cells: STAT6 is required for Th2 development and memory, while STAT4 (activated by IL-12) promotes Th1 differentiation. STAT6 acts not only in immune cells but also in lung parenchymal cells (*e.g.* epithelial cells), where it drives mucus cell changes and the recruitment of Th2 cells by regulating chemokines such as eotaxin [46].

#### Mitogen-activated protein kinase signalling cascade

Within the mitogen-activated protein kinase (MAPK) class, the p38 subtype is the most significantly associated with pneumonitis. Various environmental factors, including aeroallergens, tobacco smoke, atmospheric pollutants and respiratory microbes, activate the p38α isoform, which subsequently upregulates the expression of an array of proinflammatory cytokines and chemokines, as well as the synthesis of certain fibrogenic factors. Consequently, p38 MAPK-mediated bronchial inflammation and remodelling are instrumental in the initiation, maintenance and aggravation of airflow obstruction, a hallmark of asthma. Eosinophilic asthma emerges from both atopic and non-atopic mechanisms, predominantly influenced by Th2 lymphocytes and group 2 innate lymphoid cells (ILC2). Th2 and ILC2 cells secrete substantial quantities of IL-4, IL-5 and IL-13, which are instrumental in IgE production, eosinophilic inflammation and airway hyperreactivity, respectively. Th2 lymphocytes are activated by IL-4, whereas ILC2 are stimulated by cytokines from the innate immune response, notably thymic stromal lymphopoietin (TSLP), IL-25 and IL-33. The p38 mitogen-activated protein kinase (MAPK) pathway is instrumental in mediating both the differentiation and activation processes of Th2 cells, consequently promoting the secretion of Th2 cytokines, specifically IL-4, IL-5 and IL-13. The upregulation of gene networks associated with the p38 MAPK signalling cascade is significantly correlated with neutrophilic inflammation in bronchial tissues. Furthermore, p38 MAPK enhances the expression of intercellular adhesion molecule-1 (ICAM-1) on the endothelial cells of pulmonary vasculature and increases the secretion of tumour necrosis factor-alpha (TNF-α) from neutrophils, thereby facilitating the recruitment of these immune cells into the airway passages. Transforming growth factor beta (TGF-β) can induce apoptosis in human airway epithelial cells via p38 MAPK activation. Additionally, p38 MAPK seems to play a pivotal role in the structural modifications that underlie airway remodelling in asthma, exemplified by the hypertrophy of the sub-epithelial basement membrane. Intercellular interactions involving mast cells and lung fibroblasts undergo proliferation and secrete substantial quantities of collagen via the p38 MAPK-dependent IL-6 secretion [47].

#### Hypoxia-inducible factor-1 alpha

Key transcription factor induced by low oxygen is hypoxia-i factor-1 alpha (HIF-1α), which is upregulated under hypoxic conditions. Tumour necrosis factor-alpha (TNF-α) exerts regulatory influence on the expression levels of both the protein and mRNA of hypoxia-inducible factor 1-alpha (HIF-1α), which serves as an essential component in the functional efficacy of airway smooth muscle cells (ASMCs) and in the pathogenesis of airway inflammatory disorders. The upregulation of HIF-1α, induced by hypoxic conditions, facilitates the ubiquitination of P53 by modulating MDM2, thereby intensifying airway swelling in asthmatic conditions and promoting airway restructuring. HIF-1α is known to augment the concentrations of pro-inflammatory cytokines such as IL-4, IL-5 and IL-13. Furthermore, HIF-1α may enhance the capacity of ASMCs to endure, proliferate, migrate and elicit an inflammatory response within a hypoxic milieu, while concurrently impeding their apoptotic processes [48].

#### Notch signalling pathway

The notch signalling pathway participates in airway inflammation, airway hyperresponsiveness (AHR) and structural remodelling. It is integral to the regulation of an array of cellular processes, including proliferation, development and differentiation. Generally, the Notch signalling pathway is activated by ligands that associate with their specific receptors. The Notch signalling pathway is instrumental in the differentiation of T helper 1 (Th1) and T helper 2 (Th2) cells and has the potential to influence asthma by modulating the Th1/Th2 cell equilibrium: Notch ligand delta is predominantly linked with Th1 cells, whereas Jagged Notch ligands exhibit a stronger association with Th2 cells. The manifestation of allergic asthma is mitigated by downregulating Notch-1 and upregulating Jagged 1 and 2, resulting in reduced Th2 and Th17 cytokine concentrations while increasing interferon-gamma (IFN-γ) levels. A balance between Th1 and Th2 subsets is also present, wherein Th1 cells express the transcription factor T-bet and secrete cytokines such as IFN-γ and tumour necrosis factor-alpha (TNF-α), which are involved in the cellular immune response against viral and bacterial pathogens. Conversely, Th2 cells express the transcription factor GATA-3 and secrete cytokines, including interleukin-4 (IL-4), IL-5 and IL-13, that enhance humoral immunity following pathogen invasion. The cytokine IFN-γ inhibits Th2 differentiation and function, while the cytokines IL-4 and IL-10 inhibit Th1 differentiation and function. The Notch signalling pathway has been identified as playing a significant role in CD8+ T cell-mediated AHR and inflammatory responses, with Delta1 emerging as a pivotal regulator of allergic airway inflammation. Furthermore, IL-17A, produced by Th17 cells, can directly induce Airway Hyper Responsiveness (AHR) by acting on smooth muscle within the airways. It has been elucidated that the interaction between the Notch ligand on CD4+ T cells and Jagged1 on antigen-presenting cells (APCs) is critical for Th2 cell differentiation and the initiation of IL-4 production, which further promotes AHR and airway inflammation. NOTCH3 modulates the expression of MUC5AC, a mucin protein that is expressed at elevated levels in airway epithelial cells and constitutes a primary component of airway mucus [49].

Interactions and crosstalk among these pathways enhance airway inflammation and hyperresponsiveness and network pharmacology explains how multi-component herbal compounds can simultaneously modulate these pathways, providing mechanistic insight into the therapeutic advantages over single-target drugs.

## Application of network pharmacology in asthma management

Network pharmacology integrates computational and experimental data to reveal the multi-component, multi-target mechanisms of herbal formulations or conventional drugs in asthma (Table 1). It constructs compound-target and PPI networks, uncovering complex interactions among herbal ingredients and asthma-related targets such as IL-6, TNF, IL4, EGFR and HIF1A. It also helps reveal key signalling pathways involved in asthma pathogenesis and treatment, such as PI3K/AKT, MAPK, JAK-STAT, IL-17, TNF and Th17 cell differentiation pathways, thereby showing multi-target and multi-pathway modulation. Molecular docking and dynamics simulations complement network pharmacology by validating the binding affinities and stabilities of active compounds for core asthma targets, thereby supporting proposed mechanisms. Integration with experimental validation (*in vitro*, *in vivo*) demonstrates that network pharmacology-predicted compounds reduce airway inflammation and mucus hypersecretion and modulate immune responses, thereby confirming therapeutic effects. It facilitates discovery and mechanistic elucidation of multi-component herbal medicines, advancing traditional treatments toward modern evidence-based asthma therapy [7,20,24].

**Table 1.** Herbal plants and their mechanism of action

Scientific name	Common name	Family	Mechanism of action	Figure	Ref.
*Acacia nilotica*	Gum arabic tree	Fabaceae	The attenuation of the p13AKT signalling cascade, which may play a significant role in alleviating asthma and a multitude of other inflammatory disorders	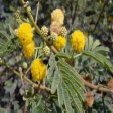	[7]
*Pinellia ternata*	BanXia	Araceae	Immune response, cytokine signalling, inflammation, reduced allergic responses and downregulating the expression of pivotal inflammatory mediators Mmp2 and IL-4 in lung tissue	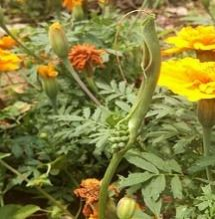	[8]
*Ferula asafoetida*	Devil's dung, hing	Umbelliferae	Airway inflammation, remodelling and immune responses	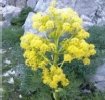	[38]
*Nepeta bracteata*	Bracted catmint	Lamiaceae	Regulating inflammation, oxidative stress and disturbed metabolic pathways	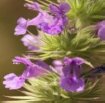	[40]
*Fructus Xanthii*	Cocklebur fruit, xanthium fruit, siberian cocklebur	Asteraceae	*Fructus Xanthii* demonstrates anti asthmatic properties through the modulation of HSP90AB1/IL6/TNF and PI3K-AKT signalling cascade, thereby influencing inflammation, cell cycle dynamics, apoptosis and the maintenance of immune homeostasis	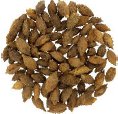	[50]
*Marsdenia tenacissima*	Rajmahal hemp, devil's tongue	Apocynaceae	*Marsdenia tenacissima* administers treatment for NA via the IL-6/JAK/STAT and PI3K/AKT/mTOR signalling pathways	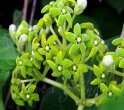	[51]
*Paeoniae radix alba*	White peony root	Paeoniaceae	Inhibit airway eosinophil infiltration, inflammatory mediator release, attenuates adipocyte lipolysis. Helped relax bronchial smooth muscle by regulating calcium ion signalling, thereby potentially reducing cough and bronchospasm.	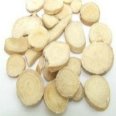	[52]

### Network pharmacology evidence for anti-asthmatic agents

#### Bushenyiqi decoction

This investigation examined the therapeutic mechanisms of BYD-a contemporary formulation of TCM for the management of allergic asthma-using network pharmacology, molecular docking and empirical validation *in vivo*. The analysis conducted via network pharmacology identified 116 bioactive compounds in BYD from the TCMSP database [26], of which 11 key constituents (notably quercetin, kaempferol and luteolin) were most strongly associated with asthma-related pathways. From the intersection of 166 BYD targets and 1,485 asthma-related genes sourced from GeneCards and OMIM [31,33] 75 genes exhibiting overlap were identified. The analysis of the protein-protein interaction (PPI) network was performed using Cytoscape [53], which elucidated IL6, EGFR, HIF1A, HSP90AA1, MAPK8, BCL2, CASP3, MYC and ESR1 as pivotal targets. The subsequent GO and KEGG enrichment analyses indicated that the BYD-asthma targets predominantly focused on PI3K/AKT, TNF, Th17 cell differentiation, HIF-1 and IL-17 signalling pathways, and consistently pointed to the PI3K/AKT signalling pathway as the central mechanism underlying BYD’s anti-asthmatic effects. Molecular docking through Autodock software [41] further confirmed strong binding affinities between the core compounds (quercetin, kaempferol, luteolin) and the core targets (IL6, EGFR, HIF1A), supporting their mechanistic relevance. *In vivo* experiments using an Ovalbumin-induced allergic asthma mouse strain demonstrated that BYD significantly reduced airway hyperresponsiveness, inflammatory cell infiltration, Th2 cytokines (IL-4, IL-5, IL-13), mucus hypersecretion and collagen deposition. Importantly, Western blot analysis showed that BYD markedly suppressed the phosphorylation of PI3K and AKT, confirming downregulation of the PI3K/AKT pathway in lung tissues. Overall, the study concludes that BYD exerts multi-component, multi-target and multi-pathway therapeutic effects against asthma, primarily by inhibiting PI3K/AKT signalling and consequently reducing airway inflammation and remodelling. These findings provide modern scientific evidence supporting the traditional "Bushenyiqi" therapeutic concept for the management of asthma [24].

#### Danlong Dingchuan decoction

The study investigated the therapeutic mechanisms of Danlong Dingchuan decoction (DLDD), a traditional Chinese medicine formula, in asthma by integrating network pharmacology, molecular docking, metabolomics and *in vivo* experiments. A total of 247 active phytoconstituents were found from the TCMSP database and 155 asthma-related targets were identified through OMIM [26,33], TTD and other databases, with quercetin, kaempferol, β-sitosterol, xanthine, lysine and luteolin emerging as key components, alongside IL-6, TNF, CXCL8, VEGFA, MAPK3, IL-1β, IL-4 and TLR4, have been delineated as pivotal therapeutic targets. The results of Gene Ontology (GO) and Kyoto Encyclopedia of Genes and Genomes (KEGG) [22,23] enrichment analyses indicated that DCDD may exert anti-asthmatic effects primarily by modulating the cAMP, cGMP-PKG, NF-κB and PI3K-Akt signalling pathways. Molecular docking confirmed high-affinity binding, particularly between quercetin and targets such as TNF, CXCL8 and TLR4. Animal experiments in ovalbumin-induced asthmatic mice demonstrated that DCDD effectively reduced airway inflammation, lowered levels of inflammatory mediators including TNF-α, IL-4, IL-6, IL-8, IL-1β and TGF-β1, and downregulated TLR4 mRNA expression, supporting its role in regulating Th2-mediated inflammation and alleviating asthma pathology. Overall, the findings highlight DLDCD as a multi-constituent, polytarget and multi-pathway intervention with both immunomodulatory and anti-inflammatory benefits in asthma [42].

#### Conciliatory anti-allergic decoction

The study investigates the therapeutic mechanisms of a multi-herb decoction used in Traditional Chinese Medicine for asthma by integrating network pharmacology, molecular docking and laboratory confirmation. Conciliatory anti-allergic decoction (CAD) comprises 10 herbs with 77 active ingredients screened from the TCMSP database according to the ADME criteria targeting 48 asthma-associated proteins taken from TTD, OMIM and PharmGKB databases, including TNF, IL4, IL5, IL10, IFN-gamma and IL13. Network pharmacology coupled with molecular docking indicated significant involvement of Th1/Th2 differentiation, NF-κB, IL-17, JAK-STAT and T cell receptor signalling pathways. Molecular docking confirmed strong binding affinities between key phytochemicals and hub targets, suggesting potent regulatory interactions. *In vivo* mouse models confirmed CAD’s efficacy in reducing airway inflammation, goblet cell hyperplasia, mucus secretion and pro-inflammatory cytokines. Major bioactive such as quercetin and kaempferol target multiple cytokines to restore immune balance. Overall, the findings demonstrate that the multi-herb decoction acts through a multi-component, multi-target and multi-pathway mechanism to reduce airway inflammation, modulate immune responses and improve asthma outcomes, supporting its relevance as a complementary therapeutic option [43].

#### Maxing-Ganshi decoction

The study systematically investigates the mechanisms of Maxing Ganshi decoction (MXGSD), a classical four-herb formula (Mahuang, Xingren, Gancao, Shigao), in the treatment of asthma using a network pharmacology approach. From 600 known herbal constituents, 141 bioactive components were identified and linked to 186 putative targets, of which 52 overlapped with asthma-related genes. Functional enrichment of these shared targets indicated involvement in airway inflammation, airway remodelling, immune regulation and drug-responsiveness pathways, aligning with known asthma pathophysiology. Construction of a comprehensive PPI network followed by topological filtering revealed 138 core targets, which were clustered into functional modules associated with gene expression regulation, DNA/RNA repair, protein synthesis, inflammatory signalling and neuro-immune interactions. KEGG pathway analysis highlighted four major signalling routes-neurotrophin, estrogen, PI3K-Akt and ErbB pathways as central mechanisms through which MXGSD may exert multi-component and multi-target therapeutic effects in asthma. Overall, the findings suggest that asthma is a systemic neuro-immuno-inflammatory disorder and that MXGSD treats the disease holistically by modulating interconnected genetic and signalling networks rather than isolated pathways, providing a basis for future drug development and validation [54].

#### Jiegeng decoction

The study examines the therapeutic potential and mechanisms of Jiegeng decoction (JGT), a traditional Chinese herbal formula composed of *Platycodon grandiflorus* and *Glycyrrhiza uralensis*, in treating allergic asthma. JGT helps improve lung function, clears mucus and reduces inflammation. Using an ovalbumin-induced mouse model, researchers demonstrated that JGT markedly reduced airway inflammation, eosinophil infiltration, Th2-associated cytokines (IL-4, IL-5, IL-13) and serum IgE levels. LC-MS analysis identified 38 phytochemical constituents in JGT. Network pharmacology analysis revealed 320 therapeutic targets, of which 54 core targets were primarily involved in immune regulation and inflammatory pathways. Enrichment analysis highlighted Th2 differentiation and the JAK-STAT signalling axis as key pathways influenced by JGT. Experimental validation (qPCR, flow cytometry, Western blotting) confirmed that JGT inhibits Th2 cell differentiation by suppressing the JAK1-STAT6 pathway in CD4⁺ T cells. Overall, the study provides mechanistic evidence that JGT ameliorates allergic asthma by modulating immune responses, particularly by blocking the IL-4/IL-13-JAK1-STAT6 signalling cascade, offering a promising multi-target herbal strategy for allergic asthma management [55].

#### Artesunate

This article provides a comprehensive network pharmacology and molecular docking analysis to evaluate the efficacy and safety of artesunate, derived from *Artemisia annua*, and its active metabolite, dihydroartemisinin (DHA), which show acceptable drug-likeness and safety profiles, as assessed by SwissADME and ADMETlab, for the treatment of asthma. Artesunate, a semisynthetic derivative of artemisinin, has exhibited therapeutic potential, including reversal of bronchial hypersensitivity and steroid resistance, attenuation of inflammation, inhibition of mast cell mediator release and promotion of eosinophil apoptosis. A comprehensive total of 282 targets associated with artesunate/DHA were extracted from the SwissTargetPrediction and PharmMapper databases, while for asthma, 7,997 targets were obtained from the GeneCards and DisGeNET databases. The investigation revealed 172 common targets shared between artesunate/DHA and asthma-related genetic loci, comprising 10 pivotal hub genes, namely CCND1, CASP3, MTOR, ERBB2, MAPK3, EGFR, MAP2K1, PTGS2, JAK2 and CASP8, which were subsequently visualized using Cytoscape software. Enrichment analyses indicated the involvement of pathways related to steroid hormone biosynthesis and metabolism, immune and inflammatory responses, AHR, airway remodelling and regulation of cell survival and apoptosis. Molecular docking confirmed stable interactions between artesunate/DHA and most hub proteins, including PTGS2, MAPK3, JAK2 and mTOR, supporting multiple mechanisms by which these compounds may alleviate asthma pathophysiology. Artesunate modulates airway smooth muscle proliferation, inflammation and glucocorticoid sensitivity, validated by bioinformatics and preclinical data, making it a potential multipotent anti-asthmatic agent [6].

### Acacia nilotica

This study investigates the anti-asthmatic potential of *Acacia nilotica* using a network pharmacology and molecular docking approach. From an initial set of 51 phytochemicals identified in AN, 18 active compounds were selected through ADMET screening. These compounds exhibited good oral bioavailability, skin permeability, BBB/CNS penetration and non-toxicity. Target prediction using the STITCH and BindingDB databases identified 189 constituent-related genes and 2096 asthma-related genes from the DisGeNet database, with 80 overlapping targets identified as potential AN-anti-asthmatic targets. Construction of a compound-target network showed that apigenin and quercetin were the major active constituents with the highest connectivity, each interacting with 35 asthma-related genes. PPI analysis delineated AKT1, EGFR, VEGFA, RELA, ESR1, HDAC1, STAT1, PPARG, AR and HSP90AB1 as pivotal hub genes. GO enrichment analysis underscored their involvement in the regulation of inflammation, hypoxia response and signalling through protein kinase B. KEGG pathway analysis indicated that the PI3K-AKT signalling pathway was the most significantly enriched pathway, suggesting a fundamental mechanism underlying the anti-asthmatic effects of AN. Other significant pathways encompassed MAPK and Ras signalling. To elucidate the mechanism by which AN operates in the context of asthma, network analysis was performed using DAVID and Cytoscape, which identified 18 active components, 80 prospective targets and 10 essential pathways. Apigenin and quercetin showed strong binding affinities to major target proteins (AKT1, EGFR, VEGFA, HSP90AB), indicating their therapeutic relevance. The findings suggest that AN exerts multi-target, multi-component and multi-pathway effects, mainly through immunomodulatory and anti-inflammatory mechanisms. The authors conclude that AN has promising anti-asthmatic potential by modulating pathways central to airway inflammation and remodelling, particularly the PI3K-AKT pathway. However, they note that further *in vivo* and *in vitro* validation is required to confirm these computational predictions [7].

### Pinellia ternata

This article presents a network pharmacology-based investigation into the mechanisms by which *Pinellia ternata* (PT), a traditional Chinese medicinal herb, exerts therapeutic effects in asthma. The study combines computational predictions with experimental animal validation to clarify PT’s bioactive components, molecular targets, affected pathways and functional effects in the context of allergic asthma. The authors identified 11 bioactive compounds in PT (mainly sterols, flavonoids and glycosides) with favourable pharmacokinetic profiles using the TCMSP Database. Targets of PT’s compounds were predicted using PharmMapper [29] and compared with asthma-related targets collected from the OMIM database, revealing 57 hub genes central to the putative pharmacological network. GO and KEGG pathway enrichment analyses showed these hubs are primarily involved in immune response, cytokine signalling and inflammation, with key signals mediated via the JAK-STAT pathway, T-cell receptor pathway and cytokine-receptor interactions. Animal experiments using an ovalbumin-induced allergic asthma mouse model revealed that PT treatment significantly reduced allergic responses, in part by decreasing Th2 cell activation and downregulating the expression of pivotal inflammatory mediators, Mmp2 and IL-4 in lung tissue. PT appears to modulate core asthma pathways, specifically by impacting cytokine signalling and matrix remodelling, thus providing a scientific rationale for its clinical application as a complementary or adjunct asthma therapy. These findings support the utility of network pharmacology for dissecting the molecular basis of complex herbal medicines and for driving forward rationale phytopharmacological drug development [8].

### Hyssopus cuspidatus Boriss

This article presents a network pharmacology investigation aimed at elucidating the antiasthmatic mechanisms of *Hyssopus cuspidatus Boriss*. (Shen Xiang Cao, SXC), a traditional herbal remedy employed by ethnic minorities in Xinjiang, China. The researchers discerned eight bioactive compounds from SXC *via* a comprehensive literature review and the TCMSP database [26], employing criteria based on oral bioavailability and DL score and subsequently predicted 258 potential human target proteins associated with asthma utilizing SwissTargetPrediction. A cumulative total of 1295 asthma-related intersection targets were extracted from DisGeNET, GeneCards and the Herb database. Using a Venn diagram, 135 common genes were identified between SXC and asthma. Notable hub targets included inflammatory cytokines and regulatory proteins, such as IL-6, TNF, IL-10, JUN and CXCL8. Functional enrichment analysis indicated that SXC predominantly influences biological processes pertinent to oxidative stress, reactive oxygen species and immune responses. Significant signalling pathways affected by SXC included MAPK, IL-17, Toll-like receptor (TLR) and TNF pathways, all of which are recognized contributors to the pathogenesis of asthma. Molecular docking studies further illustrated robust binding affinities between principal active compounds (quercetin, luteolin, acacetin and β-sitosterol) and these crucial asthma-related target proteins (TNF, MMP9 and AKT1). The study concludes that SXC manifests anti-asthmatic properties through the action of multiple compounds targeting various pathways, thereby providing scientific substantiation for its traditional application and serving as a basis for future experimental validation and clinical research [20].

### Bailing capsule (Cordyceps-based)

Network pharmacology identifies 7 key active ingredients, including cerevisterol and beta-sitosterol, and 294 overlapping targets, including asthma-targeting proteins SRC, STAT3 and TP53. These targets play a pivotal role in inflammatory signalling cascades, including the PI3K-Akt, MAPK and Ras pathways, which are primarily implicated in inflammatory responses, programmed cell death and responses to xenobiotic stimuli. The capsule exhibits anti-inflammatory effects by downregulating inflammatory mediators, including IL-4, TNF-α and IL-6. Molecular docking and molecular dynamics simulations show cerevisterol binds stably to core targets. This suggests Bailing Capsule's efficacy in modulating airway inflammation and immune responses in asthma [36].

### Ferula asafoetida

The study investigates the anti-asthmatic potential of *Ferula asafoetida* using an integrated network pharmacology and molecular docking strategy to elucidate its underlying molecular mechanisms. Among the 63 phytochemicals examined, six principal bioactive constituents, assafoetidin, cynaroside, farnesiferol-B, farnesiferol-C, galbanic acid and luteolin, were discerned based on their advantageous pharmacokinetic attributes (ADMET). The STITCH and Swiss Target Prediction databases revealed a total of 630 prospective target genes for the six bioactive compounds, while an aggregate of 1593 genes associated with asthma was obtained from the GeneCards and DisGeNET databases [31,32]. These compounds were predicted to interact with 177 asthma-associated targets through a Venn diagram. PPI network analysis highlighted AKT1, MAPK3 and TNF as central hub genes involved in airway inflammation, remodelling and immune responses. GO and KEGG enrichment revealed that the therapeutic effects of *F. asafoetida* are mainly associated with modulation of the PI3K-Akt, MAPK, NF-κB, chemokine signalling and T-cell receptor pathways, key routes implicated in asthma pathophysiology. Molecular docking further validated strong binding affinities between the six active compounds and core targets, supporting their mechanistic relevance. In summary, the results indicate that *F. asafoetida* manifests its anti-asthmatic properties through a complex mechanism involving multiple components, targets and pathways, thereby establishing a scientific rationale for its historical use and laying the groundwork for subsequent *in vitro* and *in vivo* investigations [38].

### Nepeta bracteata

The study investigated the anti-asthmatic potential of *Nepeta bracteata* (DBJJ) using an integrated metabolomics and network pharmacology approach. In an ovalbumin-induced allergic asthma rat model, DBJJ treatment significantly improved lung histopathology, reduced eosinophils and WBCs in BALF, and lowered key inflammatory mediators, including TNF-α, IL-18, IgE, IL-1β, VEGF-A and TGF-β1. Metabolomic profiling identified 21 differential serum metabolites involved in amino acid and energy metabolism, suggesting DBJJ’s role in restoring metabolic disturbances associated with asthma. UPLC-QE-MS/MS analysis identified 29 chemical constituents, of which 13 were active based on oral availability and Caco-2 cell permeability, mapping to 120 core targets and 173 KEGG pathways, including MAPK, PI3K-Akt, Th17 differentiation and oxidative stress-related pathways. Integrated analysis highlighted ferulic acid and ursolic acid as key bioactive compounds targeting DAO and NOS2 and molecular docking confirmed strong binding affinities. Western blot validation showed that DBJJ reduced phosphorylation and expression of NOS2, MAPK1/3 and STAT3, confirming pathway-level modulation. Overall, the findings demonstrate that *N. bracteata* exerts multi-target, multi-pathway anti-asthmatic effects by regulating inflammation, oxidative stress and disturbed metabolic pathways [40].

#### Resveratrol

This study employs a network pharmacology and molecular docking approach combined with empirical verification to explore the therapeutic mechanisms of resveratrol in asthma treatment. After intersecting the 236 Res targets obtained from TCMSP, DrugBank and SwissTargetPrediction with the 2,382 asthma targets from DisGeNET, GeneCards and TTD, 120 overlapping genes against asthma were identified, with TNF, IL6, STAT3, TP53 and IL1B as key therapeutic targets. Functional enrichment analyses elucidated the role of resveratrol in various biological processes, including apoptosis and inflammation, as well as in specific signalling pathways such as the TNF and MAPK pathways. Docking studies corroborated the presence of robust binding affinities between resveratrol and critical proteins (TNFα, IL-6, STAT3, p53 and IL-1β). Experimental validation using a house dust mite-induced asthma murine model and airway epithelial cell cultures demonstrated that resveratrol significantly mitigated airway hyperresponsiveness and inflammation by reinstating the dysregulated expression of these targets, with notable improvements in inflammatory infiltration in the peri-bronchial regions, peri-vascular areas and lung parenchyma. This investigation offers a thorough understanding of the multi-targeted anti-asthmatic effects of resveratrol and substantiates its prospective application in asthma management [44].

### Fructus Xanthii

This study integrates multi-omics (from GEO datasets GSE63142 and GSE14787), network pharmacology, machine learning (RF, SVM, XGBoost), molecular docking, dynamics simulations, immune infiltration (CIBERSORT) and *in vivo* validation in an OVA-induced murine asthma model to reveal *Fructus Xanthii*’s (Cang-Er-Zi) anti-asthmatic mechanisms. Key findings include 3,755 asthma-related DEGs with the MEblack WGCNA module (741 genes) correlating strongly; 1,317 Fructus Xanthii targets intersecting 100 DEGs, yielding hub genes like HSP90AB1, CCNB1, CASP9, CDK6, NR3C1, ERBB2 and CCK via PPI and machine learning, indicating anti-inflammatory, immune-modulatory, cell cycle and apoptosis-regulatory involvement. GO/KEGG enrichment highlighted steroid hormone responses, kinase activities, PI3K-AKT, cellular senescence and p53 pathways; docking showed strong affinities (*e.g.* carboxyatractyloside-HSP90AB1 at -42.22 kJ mol^-1^ (-10.09 kcal mol^-1^)) with stable MD trajectories (RMSD <0.3 nm); immune analysis revealed shifts in M2 macrophages and plasma/ /memory B cells. *In vivo*, *Fructus Xanthii* extract (10 to 30 mg kg^-1^) dose-dependently reduced lung inflammation, BALF cytokines (TNF-α, IL-6, IL-1β, IL-5), histopathology scores and hub gene/protein expression (HSP90AB1, AKT1), comparable to dexamethasone, modulating inflammation, cell cycle, apoptosis and immune homeostasis via HSP90AB1/IL6/TNF and PI3K-AKT pathways. Overall, *Fructus Xanthii* exhibits a robust multi-target therapeutic mechanism, supporting its potential as an effective complementary herbal intervention in asthma [50].

### Marsdenia tenacissima

This study investigated the therapeutic potential of *Marsdenia tenacissima* (MT) in neutrophilic asthma (NA), a severe asthma subtype characterized by poor glucocorticoid response and elevated neutrophil levels. By employing a comprehensive methodology that integrates network pharmacology, molecular docking, molecular dynamics and experimental validation, the research successfully identified significant bioactive constituents of MT, notably 17β-Tenacigenin B, as the primary pharmacologically active C21 steroid compound that interacts with IL-6 and JAK1, thereby modulating the JAK-STAT signalling pathway and the subsequent PI3K-AKT-mTOR cascade. From 282 MT compounds, 45 drug-like actives intersected with asthma targets yielded 34 genes enriched in leukocyte activation and migration, LPS response, lipoxygenase activity and relevant KEGG pathways; molecular docking showed strong binding affinities (-24.69/-25.94 kJ mol^-1^ (-5.9/-6.2 kcal mol^-1^)), confirmed by dynamics simulations (stable RMSD ~0.2 nm) and CETSA (higher Tm). Validation via molecular docking, dynamics simulations, CETSA (cell thermal shift assay), *in vitro* assays on bronchial epithelial cells showing reduced inflammatory cytokines (IL-1, IL-6, IL-17, IFN-γ, TNF-α) and neutrophil migration, and *in vivo* NA mouse models (HDM+LPS-induced) demonstrating alleviated airway hyperresponsiveness, neutrophil infiltration, mucus hypersecretion and cytokine levels. MT and 17-Tenacigenin B treatments mirrored the effects of IL-6 inhibitor LMT-28, confirming the IL-6/JAK-STAT axis as central to MT's therapeutic mechanism. Overall, this work demonstrates that *Marsdenia tenacissima* exerts potent therapeutic effects in neutrophilic asthma by modulating inflammatory signalling cascades, restoring immune balance and overcoming steroid resistance, positioning it as a promising candidate for refractory asthma management [51].

### Paeoniae Radix Alba

*Paeoniae Radix* Alba (PRA), the root of *Paeonia lactiflora* Pall., is traditionally used in Chinese medicine for asthma treatment. This study employed a systematic network pharmacology approach to identify 21 active phytochemicals in PRA and their 147 associated target proteins relevant to asthma. Key active compounds such as kaempferol, paeoniflorin, beta-sitosterol and sitogluside were linked to critical asthma-related targets, including TNF-α, PGR and NF-Κb pathway components. Gene Ontology and KEGG pathway analyses suggested that PRA’s therapeutic effects involve modulation of inflammatory processes, inhibition of TNF release, ceramide signalling, and regulation of apoptosis. Notably, kaempferol was found to inhibit airway eosinophil infiltration, inflammatory mediator release, IL-4 and ceramide signalling pathway, while Paeoniflorin diminishes lipolytic activity in adipocytes and obstructs the phosphorylation of ERK, JNK, and IKK that is induced by TNF-α. Beta-sitosterol and sitogluside helped relax bronchial smooth muscle by regulating calcium ion signalling, thereby potentially reducing cough and bronchospasm. The study highlights TNF-α as a central target, providing a molecular basis for PRA’s anti-inflammatory and immunomodulatory effects in asthma. Overall, the findings support PRA’s multi-target therapeutic potential, uncovering molecular interactions that warrant further experimental validation and clinical exploration [52].

#### Andrographolide

This research meticulously investigated the anti-asthmatic mechanisms of andrographolide (AG), a principal bioactive compound derived from *Andrographis paniculata*, which is widely recognized for its significant anti-inflammatory and immunomodulatory properties, as well as its ability to restore steroid sensitivity. Employing a combination of network pharmacology, molecular docking and experimental validation within a model of ovalbumin-sensitized BALB/c mice, the study identified a total of 57 andrographolide-associated targets, sourced from the Swiss Target Prediction, Drug Bank and STITCH [28,30,34] databases, which were subsequently cross-referenced with 8168 asthma-related targets obtained from the OMIM [31] and Genecards 33] databases. A subset of 38 targets emerged as potential candidates for andrographolide's therapeutic action against asthma. The construction of a PPI network underscored prominent hubs, including IL-6, IL-1B, NFKB1, MMP9, CDK2, CREBBP, MAP2K1, JAK1, AR and PRKCA. GO enrichment analysis highlighted critical biological processes such as protein phosphorylation and kinase activity, along with cellular components like receptor complexes, while KEGG pathway analysis identified Th17 cell differentiation, JAK-STAT signalling, PI3K-Akt pathway and TNF signalling as the primary pathways of interest. Molecular docking studies corroborated the strong binding affinities of AG to various targets, including JAK2 (-21.30 kJ mol^-1^), MMP9 (-21.63 kJ mol^-1^), PRKCA (-21.71 kJ mol^-1^), LRRK2 (-21.92 kJ mol^-1^) and ITGAL (-23.35 kJ mol^-1^), with interactions visualized through the formation of hydrogen bonds. *In vivo* flow cytometry experiments demonstrated that AG (0.5 mg kg^-1^) significantly reduced Th17 cell differentiation in lung tissue, comparable to dexamethasone (2 mg kg^-1^), supporting Th17 inhibition as a core mechanism for AG's therapeutic effects against airway inflammation, hyperresponsiveness and remodelling in asthma [56].

Collectively, the body of research elucidates those herbal preparations and their constituent phytochemicals manifest anti-asthmatic properties through multifaceted mechanisms encompassing multiple components, targets and pathways, with a predominant emphasis on the modulation of inflammatory, immunological and airway remodelling pathways. Most traditional decoctions, such as Danlong Dingchuan Decoction, Bushenyiqi Decoction, Maxing-Ganshi Decoction and Jiegeng Decoction, consistently targeted central asthma-related pathways, including PI3K-Akt, MAPK, NF-κB, JAK-STAT, TNF and Th2/Th17 differentiation axes. Similarly, single herbs or phytochemicals such as *Acacia nilotica*, *Pinellia ternata*, *Ferula asafoetida*, *Hyssopus cuspidatus* Boriss., Resveratrol, *Paeonia lactiflora* and Andrographolide showed overlapping hub targets such as TNF, IL-6, AKT1, MAPK3, STAT3 and EGFR, reinforcing the concept that asthma is driven by interconnected inflammatory and immune signalling networks. Across studies, network pharmacology combined with molecular docking served as the foundational methodology, while several investigations strengthened predictions through *in vivo* validation in ovalbumin, HDM, or LPS-induced asthma models. Among them, studies integrating multi-omics, machine learning, molecular dynamics, immune infiltration analysis and experimental validations, such as those on *Fructus Xanthii* and *Marsdenia tenacissima*, provided the most comprehensive mechanistic insight, as they moved beyond static network predictions to dynamic simulations and subtype-specific validation (*e.g.* neutrophilic asthma). Therefore, while classical network pharmacology with docking and animal validation is robust and widely applied, integrative approaches incorporating multi-omics data, machine learning algorithms, molecular dynamics simulations and targeted experimental confirmation appear methodologically superior, offering higher predictive accuracy, more profound mechanistic clarity and stronger translational relevance for future anti-asthmatic drug development (Tables 2 and 3).

**Table 2.** Summary of network pharmacological studies on herbal compounds for asthma

No.	Formula/component	Key bioactive	Main targets/pathways	Study design and experimental model	Ref.
1	Artesunate	Artesunate/DHA	CCND1, MTOR, ERBB2, MAPK3, EGFR, NF-kappa B signalling pathway, Fc epsilon receptor (FCERI) signalling	Network pharmacology, ADMET and molecular docking	[6]
2	*Acacia nilotica*	Quercetin, apigenin	AKT1, EGFR, VEGFA, STAT1, HSP90AB1, MAPK, PI3K-Akt, Ras	Network pharmacology and Molecular docking (*in silico*)	[7]
3	*Pinellia ternata*	Flavonoids, sterols, lipids	JAK-STAT, TCR, cytokine-cytokine receptor, IL-4, MMP2	Animal model, gene/protein expression	[8]
4	*Hyssopus cuspidatus* Boriss.	Luteolin, quercetin, acacetin and β-sitosterol	TNF, MAPK, IL-17, MMP9, AKT1, JUN, CXCL8, IL-6, TLR	Network pharmacology and Molecular docking (*in silico*)	[20]
5	Bushenyiqi decoction	Quercetin, kaempferol, luteolin	IL-6, EGFR, HIF1A, PI3K-Akt, Th2 cytokines (IL-4, IL-5, IL-13), TNF, HIF-1, Th-17 cell differentiation	Network pharmacology, Mouse model, western blotting, ELISA, molecular docking, cytokine levels	[24]
6	Bailing capsule	Cerevisterol	SRC, TP53, STAT3, PI3K-Akt, MAPK and Ras pathways	Network pharmacology, molecular docking, *in vitro* validation, molecular dynamics simulation	[36]
7	*Ferula asafoetida*	Assafoetidin, luteolin	AKT1, MAPK3, TNF, NF-κB, PI3K-Akt, Ras, Chemokine signaling	Network pharmacology and molecular docking (*in silico*)	[38]
8	*Nepeta bracteata* (DBJJ)	Ferulic acid, ursolic acid	MAPK, STAT3, NOS2, Th17 differentiation, oxidative stress	Metabolomics, western blot, molecular docking, animal model	[40]
9	Danlong Dingchuan decoction	Quercetin, xanthine, lysine, kaempferol and ß sitosterol	IL-6, TNF, CXCL8, VEGFA, MAPK3, cAMP, cGMP-PKG, NF-κB and PI3K-Akt signalling pathway	Metabolomics, experimental validation and molecular docking, *in vivo* studies	[42]
10	Conciliatory anti-allergic decoction	Quercetin, kaempferol	TNF, IL-4, IL-13, NF-κB, JAK-STAT, Th1/Th2	Mouse model, molecular docking	[43]
11	Resveratrol	Resveratrol	TNF, IL6, STAT3, TP53, IL1B, apoptosis, MAPK, TNF signalling pathways	Animal/cell experiments, molecular docking, western blotting	[44]
12	*Fructus Xanthii*	sesquiterpene lactones, flavonoids, lignans and coumarin derivatives	HSP90AB1, CCNB1, CASP9, CCK, HSP90AB1/IL6/TNF, PI3K-AKT, cellular senescence, p53 pathways	multi-omics analysis, network pharmacology, molecular docking, machine learning and experimental validation	[50]
13	*Marsdenia tenacissima*	17-Tenacigenin B	IL-6, JAK1, JAK-STAT and PI3K-AKT-mTOR pathways	network pharmacology, molecular docking, MD and experimental validation	[51]
14	*Paeoniae radix* Alba	kaempferol, paeoniflorin, beta-sitosterol and sitogluside	TNF-α, PGR, NF-κB, ceramide signalling, Toll-like receptor pathways and apoptosis regulation	Network/enrichment analysis	[52]
15	Maxing Ganshi decoction	Multiple flavonoids	Neurotrophin, PI3K-Akt, estrogen, immune inflamemation and ErbB pathways	Network/enrichment analysis	[54]
16	Jiegeng decoction	*Platycodon grandiflorus* and *Glycyrrhiza uralensis*	IL-4/IL-13-JAK1-STAT6	Animal model, network, experimental validation	[55]
17	Andrographolide	Andrographolide	IL-6, IL-1B, NFKB1, MMP9, CDK2, Th17 cell differrentiation, JAK-STAT, PI3K-Akt and TNF signalling pathways	Network pharmacology, molecular docking, *in vitro* validation	[56]

**Table 3.** Comparative summary of studies investigating herbal/phytochemical interventions in asthma using network pharmacology and experimental models

No.	Study / herb or formula	Major strengths of the model	Key limitations of the model	Ref.
1	Artesunate	Incorporates safety and pharmacokinetic prediction; enhances drug repositioning relevance	No experimental asthma model used; therapeutic efficacy remains theoretical	[6]
2	*Acacia nilotica*	Efficient identification of multi-target mechanisms; suitable for exploring traditional herbal claims	No biological or experimental validation; predictions rely on database accuracy; limited translational relevance	[7]
3	*Pinellia ternata*	Combines computational prediction with *in vivo* validation; links molecular targets to biological outcomes; confirms anti-allergic activity	Small animal sample size; OVA model reflects acute allergic asthma rather than chronic disease; network pharmacology predictions require further clinical confirmation	[8]
4	*Hyssopus cuspidatus* Boriss.	Systematic screening of key inflammatory pathways (MAPK, TNF, IL-17)	Purely predictive; lacks functional immune or airway validation	[20]
5	Bushenyiqi decoction	Strong causal evidence; links pathway modulation to airway inflammation and lung function	Animal model reflects mainly allergic asthma; limited human translatability	[24]
6	Bailing capsule	Confirms predicted targets in airway epithelial cells, improves biological relevance	*In vitro* model cannot replicate full asthma pathology or airway remodelling	[36]
7	*Ferula asafoetida*	Highlights multi-component synergy and pathway-level modulation	Absence of *in vitro* or *in vivo* confirmation; does not account for pharmacokinetics or immune complexity	[38]
8	*Nepeta bracteata* (DBJJ)	Demonstrates direct pharmacological effect of single herb; easier to interpret than polyherbal formulas	Often lacks omics-level validation; preclinical data only; limited pathway confirmation	[40]
9	Danlong Dingchuan decoction	Demonstrates functional anti-inflammatory effects in whole-organism context	Limited mechanistic depth at cellular immune level; species differences	[42]
10	Conciliatory anti-allergic decoction	Systems-level approach suitable for complex diseases like asthma; reflects traditional polyherbal therapy	High complexity reduces clarity of causal mechanisms; dosing standardization issues	[43]
11	Resveratrol	Well-characterized compound with defined molecular targets; strong mechanistic evidence	Non-herbal single compound - may not fully represent TCM complexity; bioavailability concerns limit translational interpretation	[44]
12	*Fructus Xanthii*	Most comprehensive approach; captures immune heterogeneity and system-level effects	High complexity; resource-intensive; limited scalability for routine screening	[50]
13	*Marsdenia tenacissima*	Whole-organism assessment allows evaluation of airway inflammation and cytokine response	Limited mechanistic depth; often lacks long-term chronic remodelling assessment; translational relevance to humans may be restricted	[51]
14	*Paeoniae radix* Alba	Mechanistic insight into active constituents; clearer pharmacological targeting compared with complex decoctions	May not reflect synergistic effects seen in clinical formulas; dose equivalence to human use unclears	[52]
15	Maxing Ganshi decoction	Classical TCM formula studied in physiological disease context; multi-target approach mirrors clinical usage	Complex multi-component nature makes mechanistic attribution difficult; variability in herbal composition reduces reproducibility	[54]
16	Jiegeng decoction	High translational strength; validates Th2-JAK-STAT signalling at cellular and tissue levels	Focused on Th2-dominant asthma; may not represent non-allergic phenotypes	[55]
17	Andrographolide	Strengthened mechanistic credibility; partial immune validation (Th17 differentiation)	Limited to cellular models; lacks airway-level physiological validation	[56]

## Integration with experimental validation

The integration of network pharmacology with empirical validation enhances understanding of the mechanistic foundations underlying the therapeutic efficacy of herbal medicines in the management of asthma. For BYD, network pharmacology identified key bioactive compounds, such as quercetin and kaempferol, that target pivotal molecules, including IL-6 and EGFR, thereby modulating the PI3K/AKT signalling pathway. *In vivo* studies using ovalbumin-induced asthma models demonstrated BYD’s efficacy in reducing airway inflammation, Th2 cytokine levels (IL-4, IL-5, IL-13) and airway remodelling, supported by histological and protein expression analyses. Molecular docking analyses validated significant binding affinities between the active constituents of BYD and targets associated with asthma [4]. Similarly, *Pinellia ternata* mechanism was elucidated using network pharmacology, revealing hub targets involved in T-cell receptor and JAK-STAT pathways. Experimental validation demonstrated PT’s ability to suppress Th2 immune responses and downregulate asthma-associated genes, such as MMP2 and IL-4, in animal models, with reduced cytokine levels confirmed by ELISA. These examples exemplify how computational predictions guided targeted experimental assays, collectively validating the multi-target, multi-pathway therapeutic effects of these herbal formulations in asthma management [8]

## Challenges and limitations

**Figure 3.**
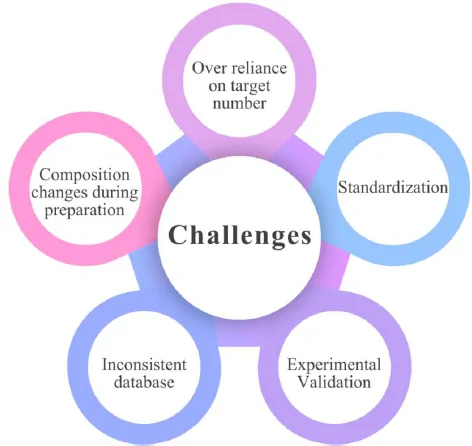
Challenges in network pharmacology

Despite the promising mechanistic insights offered by these networkpharmacology-based studies on herbal extracts in asthma, several conceptual and practical challenges remain to be addressed, both in methodology and in clinical translation (Figure 3).

Network pharmacology, despite its conceptual strengths, faces several practical and methodological challenges that limit the robustness and clinical translatability of its predictions. One major issue is the inconsistency and incompleteness of underlying databases. Because the discipline depends heavily on network and target databases for primary data, outcomes can differ substantially between studies or fail to align with experimental observations, especially when poorly curated or outdated entries are used [42].

Another critical concern is the quality and safety assessment of herbal medicines. The concentrations of active and potentially toxic constituents can vary widely across batches, regions and preparation methods, and many studies on traditional herbal formulations do not follow standardized quality-control or safety protocols. This variability complicates clinical evaluation, hinders reproducibility and weakens the reliability of networkbased predictions. Furthermore, network models often overlook systemic physiological integration, focusing on intracellular molecular networks while neglecting interorgan communication and broader regulatory systems such as the neural, immune and endocrine axes, which play central roles in diseases like asthma. The compatibility and standardization of herbal extracts pose additional hurdles. Achieving pharmaceutical-grade quality and batch-to-batch reproducibility with complex mixtures is difficult, undermining both experimental consistency and clinical relevance. At the mechanistic level, network models may also fail to account for resistance mechanisms, such as efflux transporters like Pglycoprotein, that can significantly modulate drug exposure and override predicted pharmacological effects. Moreover, many networkpharmacology studies remain heavily reliant on *in silico* outputs without adequate experimental validation; well-designed preclinical and clinical investigations are therefore essential to confirm predicted relationships between gene expression changes and actual physiological or therapeutic outcomes [56].

Methodological limitations also arise from the screening tools and criteria commonly applied. Traditional rules such as Lipinski’s Rule of Five are tailored to smallmolecule synthetic drugs and may inappropriately exclude promising herbal compounds that fall outside these narrow physicochemical ranges [56]. At the same time, current databases often ignore the actual content and dose of individual components in herbal preparations, even though efficacy depends on whether bioactive constituents reach therapeutic concentrations in target tissues. The chemical composition of herbal medicines can change during preparation (*e.g.* during decoction, influenced by time, temperature and solvent), but such variability is not reflected in existing network databases or analyses. Finally, an overreliance on target number can distort the identification of key active components, as methods that prioritize compounds with many predicted targets may overlook agents with fewer but more potent and functionally relevant interactions [50].

## Future perspectives

Despite these limitations, network pharmacology offers a promising platform for advancing the scientific understanding and clinical application of herbal medicines. A key direction is the development of more comprehensive network models that integrate intracellular signalling with extracellular crosstalk and multisystem physiology across organs. Such models would better capture the systemic nature of diseases such as asthma and reflect how herbal interventions modulate interactions among immune, respiratory and other regulatory systems.

Another important frontier is mechanistic and translational research. Well-designed preclinical and clinical studies are needed to elucidate the precise modes of action of botanical and hybrid preparations, thereby strengthening the scientific credibility of traditional herbal interventions. Emerging analytical technologies, including advanced mass spectrometry and twodimensional liquid chromatography, will play a crucial role in characterizing complex herbal mixtures and tracking their pharmacologically active components, enabling more accurate mapping of compound-target relationships.

The field is also poised to benefit from the holistic integration of multi-omics data and artificial intelligence. By combining genomics, transcriptomics, proteomics and metabolomics with machinelearning approaches, network pharmacology can more rationally decode polypharmacology, predict adverse effects and refine precisionmedicine strategies in phytomedicine. Closely linked to this is the need for rigorous experimental validation of networkbased predictions through systematic analyses of gene and protein expression in cellular and animal models, as well as in clinical settings, to bridge the gap between computational inference and biological reality [57].

In parallel with these analytical advances, the quality and safety standards for herbal and botanical medicines must be strengthened. Adoption of internationally recognized guidelines for standardization, pharmacological characterization and safety assessment will improve the reproducibility and clinical reliability of herbal interventions [58]. At the same time, existing databases should be expanded and enriched to include information on component content, dosage and the effects of different preparation methods, as well as links between compounds, targets, diseases and traditional classification systems (*e.g.* TCM syndromes). Incorporating dose-effect relationships into network models by explicitly linking herbal medicine dosages, tissue concentrations, target engagement and therapeutic outcomes would further enhance the predictive power of the approach.

Finally, the integration of molecular docking and other experimental techniques within the network--pharmacology pipeline will strengthen confidence in predicted compound-target interactions and provide deeper mechanistic insights. By combining large-scale network analysis with fine-grained structural and functional validation, future research can transform network pharmacology from a descriptive tool into a robust, hypothesis-driven framework for the rational development of herbal-based therapies for asthma and other complex diseases [58].

## Conclusion

Network pharmacology represents a transformative paradigm in asthma management, systematically elucidating the intricate, multifaceted interactions and objectives of phytotherapeutic agents to address the limitations of conventional single-target therapies, which often lead to resistance, side effects such as infections and metabolic issues and suboptimal control across heterogeneous disease phenotypes. By integrating computational predictions with experimental validation, this approach offers new opportunities to develop effective, safer and personalized anti-asthmatic therapies. This review consolidates evidence from representative studies on formulations such as Bushenyiqi decoction, Danlong Dingchuan decoction, *Acacia nilotica* and emerging ones like *Marsdenia tenacissima* and *Bacopa monnieri*, highlighting consistent modulation of pivotal pathways including PI3K-AKT, JAK-STAT, TNF, IL-17, Th17 differentiation, NF-κB and TLR/MAPK by key bioactive like quercetin, kaempferol, luteolin, stigmasterol and andrographolide, with robust validation via molecular docking, OVA-induced mouse/rat models, metabolomics, Western blots and AI-enhanced GNN frameworks that confirm reductions in eosinophil infiltration, Th2 cytokines (IL-4/IL-5/IL-13), mucus hypersecretion, neutrophil influx and airway remodelling. Despite challenges such as data heterogeneity, database limitations and the need for standardized protocols, integrative multi-omics-network approaches (*e.g.* in *Nepeta bracteata* and *Fructus Xanthii*) effectively bridge predictions to experimental outcomes, thereby minimizing discrepancies and enhancing mechanistic insights. Looking ahead, future efforts should emphasize prospective randomized controlled trials (RCTs), personalized multi-omics profiling for endotype-specific therapies, nanoparticle delivery systems for prioritized compounds (*e.g.* resveratrol, andrographolide) and AI-driven discovery to pioneer steroid-sparing, cost-effective regimens tailored to high-burden contexts like India, ultimately fostering safer, holistic, patient-centric asthma therapeutics that harness nature's polypharmacology.
